# Rehabilitation of neuromyelitis optica: Two CARE-compliant case reports

**DOI:** 10.1097/MD.0000000000017465

**Published:** 2019-10-11

**Authors:** Won Bin Kim, So Young Lee, Bo Ryun Kim, Youn Ji Kim

**Affiliations:** Department of Rehabilitation Medicine, Jeju National University Hospital, Jeju National University School of Medicine, Jeju, Republic of Korea.

**Keywords:** neuromyelitis optica, paraplegia, quadriplegia, rehabilitation

## Abstract

**Rationale:**

Neuromyelitis optica (NMO), also known as Devic syndrome, is a central nervous system demyelinating disease consisting of optic neuritis and myelitis. Several studies have reported the effects of rehabilitation programs and specific exercises on outcomes in individuals with multiple sclerosis, but few have considered individuals with NMO. We present 2 cases of paraplegia due to NMO with rehabilitation outcome.

**Patient concerns:**

The first case corresponds to a 65-year-old woman with NMO presented with C4 incomplete American Spinal Injury Association (ASIA) scale D, and the second case is a 41-year-old woman with NMO presented with C1 incomplete ASIA-C.

**Diagnoses:**

Two cases were confirmed by positive anti-aquaporin-4 antibody and presence of T2-weighted hyperintense lesion in spinal cord on magnetic resonance imaging.

**Intervention:**

The first patient planned for focusing on left hand fine motor training through occupational therapy by strengthening and stretching muscle using E-link (Biometrics Ltd, Newport, UK) during 4 weeks, and the second patient received strengthening lower extremity and gait training using a lower-body positive pressure treadmill (AlterG, Anti-Gravity Treadmill, Fremont, CA) during 4 weeks.

**Outcomes:**

After a 4-week rehabilitation, the first patient's manual muscle testing was improved to grade 2/5 to 3+/5 in left upper limb specifically. Also, Spinal Cord Independence Measure (SCIM) was improved 79 to 88. Functional gains were made in bathing, upper-extremity dressing, and using chopsticks independently. Also, the second patient's manual muscle testing improved to grades 1 to 2/5 to 3 to 4/5 generally, and ASIA scale improved C5 incomplete ASIA-D. SCIM was improved to by allowing walking independently and increasing lower-extremity dressing and toileting ability.

**Lessons:**

An intensive, multidisciplinary rehabilitation program may lead to neurological and functional gains in patients with NMO.

## 1 Introduction

Neuromyelitis optica (NMO), also known as Devic syndrome, is a central nervous system demyelinating disease consisting of optic neuritis and myelitis.^[1,2]^ These clinical manifestations can be mischaracterized as multiple sclerosis (MS). In 2006, Wingerchuk et al's^[3]^ study proposed the criteria to define the syndrome, reporting an impressive 99% sensitivity and 90% specificity. The diagnostic criteria characterize NMO by optic neuritis, myelitis, and at least 2 of the following 3 criteria must be present: longitudinally extensive cord lesion, magnetic resonance imaging nondiagnostic for MS, and NMO-IgG seropositivity.^[3–6]^ The serum autoantibody NMO-IgG, which targets aquaporin-4, is a good candidate because it is >90% specific for NMO in patients presenting with an optic-spinal syndrome and is not detected in patients with classic MS. The specificity of the antibody as a marker for NMO and its immunoreactive sites in the spinal cord occurs in NMO.^[6]^

Like MS, NMO is more frequent in women than in men.^[7]^ The onset of the syndrome varies, from young adolescence to adulthood, with a median peak incidence among individuals aged in their late 30s. In United states, NMO occurs in Caucasian individuals, but there is an over-representation among Native Americans, Hispanic Americans, Asians, and individuals of Mediterranean and African descent.^[8]^ The prevalence of MS is lower in Japan than in the Western Europe, and the ratio of classic, monophasic NMO to MS is higher.^[9]^

Several studies have reported the effects of rehabilitation programs and specific exercises on outcomes in individuals with MS, but few have considered individuals with NMO.^[10–12]^ This is most likely due to the low incidence and prevalence of NMO, and also a poor understanding of the disease. However, NMO is a progressive neurologic disease with an uncertain course, and it is important to devise a rehabilitation strategy to reduce the neurological sequelae that occur after an episode.

In this article, we present 2 cases of NMO and discuss the rehabilitation program used.

## 2 Case presentation

### 2.1 Ethical statement

This case report conforms to all CARE guidelines and reports the required information accordingly. The study was approved by the Institutional Review Board and Ethics Committee of Jeju National University Hospital. Written consent was obtained from both patients.

### 2.2 Patient 1

A 65-year-old woman was admitted to the department of rehabilitation medicine after experiencing chronic left hand dystonia and weakness for 1 year. A diagnosis of NMO was confirmed by positive anti-aquaporin-4 antibody and the presence of a T2-weighted hyperintense lesion in the spinal cord and left optic nerve on magnetic resonance images. The spinal cord lesion was at the level of C2 to C5, and was roughly 3 segment lengths in size. Neurological assessment according to the International Standards for Neurological Classification of Spinal Cord Injury (ISNCSCI)^[13]^ revealed that the patient had muscle weakness in the left upper and lower limbs (manual muscle test score of 2) and no sensory abnormality, and the American Spinal Injury Association (ASIA) classification was C4 incomplete ASIA-D.

At the time of admission to the department of rehabilitation medicine, maximal grip strength and pinch strength were measured using a dynamometer, and her grip strength was 5.4 kg for the left hand and 11.8 kg for the right hand. The box and block test score was 8 for the left hand and 21 for the right hand. The 9-hole test time was 64.3 seconds for the left hand and 32.0 seconds for the right hand. The Spinal Cord Independence Measure (SCIM) was 79, and the patient was in need of help from others to perform daily activities such as dressing or eating.

**Figure 1:**
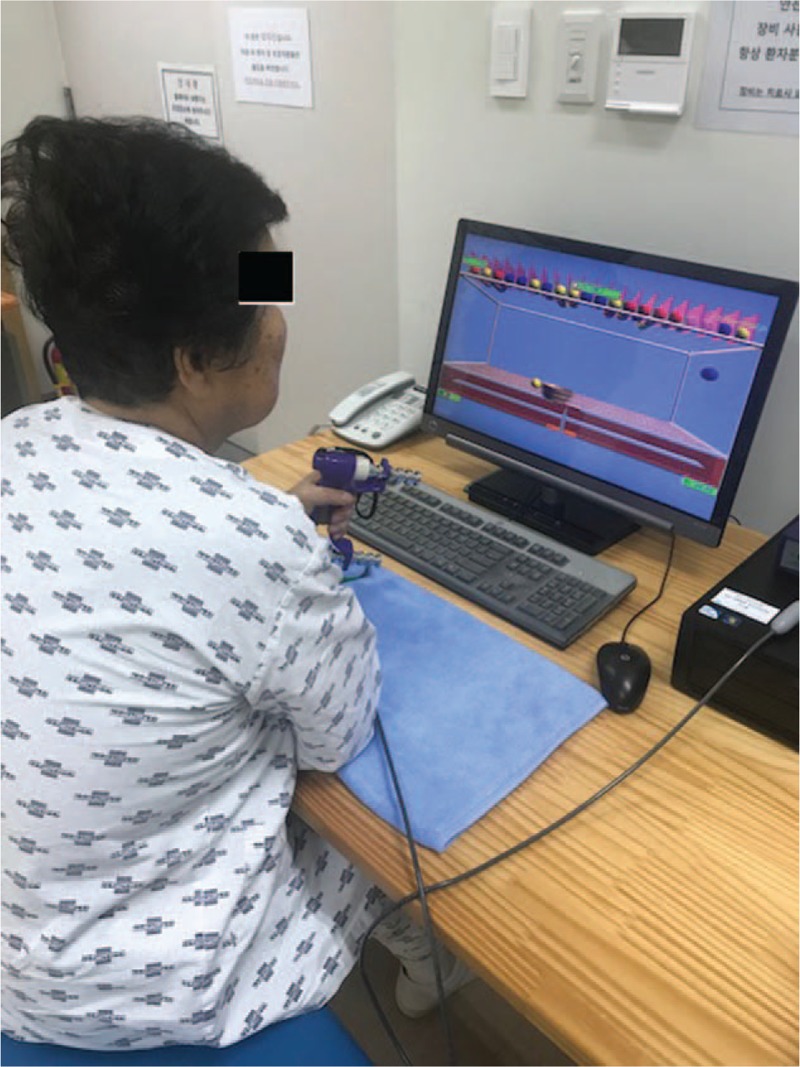
The hand fine motor training consisted of E-link (Biometrics Ltd, Newport, UK).

The patient received conventional occupational therapy focusing on upper-extremity stretching, strengthening, and performing daily activities for 30 minutes, hand fine motor training using E-link (Biometrics Ltd, Newport, UK) (Fig. 1) for 30 minutes, and physical therapy that included lower-extremity muscle strengthening and aerobic exercise for 1 hour. This rehabilitation was done 5 times a week for 4 weeks.

After 4 weeks of rehabilitation, when she was re-evaluated, manual muscle testing score in the left upper limb had improved from grade 2 to grade 3+, and the SCIM had improved from 79 to 88. Functional gains were made in all activities of daily living, especially bathing, dressing independently, feeding, and going to the toilet. Maximal grip strength and pinch strength increased to 7 kg for the left hand and 15 kg for the right hand. The box and block test improved to 12 for the left hand and was maintained at 21 for the right hand. The 9-hole test time reduced to 42.2 seconds for the left hand and 28.3 seconds for the right hand. These results indicate that fine motor control was improved and clumsiness decreased. The patient was discharged home and scheduled to attend outpatient rehabilitation twice a week.

### 2.3 Patient 2

A 41-year-old woman was admitted to the department of neurology medicine after developing weakness, an inability to independently ambulate, and impaired vision in the left eye. Two years before the admission, the patient had been diagnosed with NMO by positive anti-aquaporin-4 antibody; however, she did not take the azathioprine that had been prescribed. Neurological work-up confirmed that she had relapsed, and she was prescribed steroid pulse therapy and azathioprine. After 12 days, she was transferred to the department of rehabilitation medicine.

At the time of transfer, neurological assessment according to the ISNCSCI^[13]^ revealed that the patient had muscle weakness in the right upper and lower limbs (manual muscle test score of 2), and sensory abnormality to light touch and pinprick without a clear sensory level, and the ASIA classification was C1 incomplete ASIA-C. SCIM was 42. The patient was able to walk a short distance using a rolling walker, and the Berg balance scale score was 22. The 6-minute walk test (6MWT) distance was 110 m, and the timed up and go (TUG) test time was 31.7 seconds.

**Figure 2:**
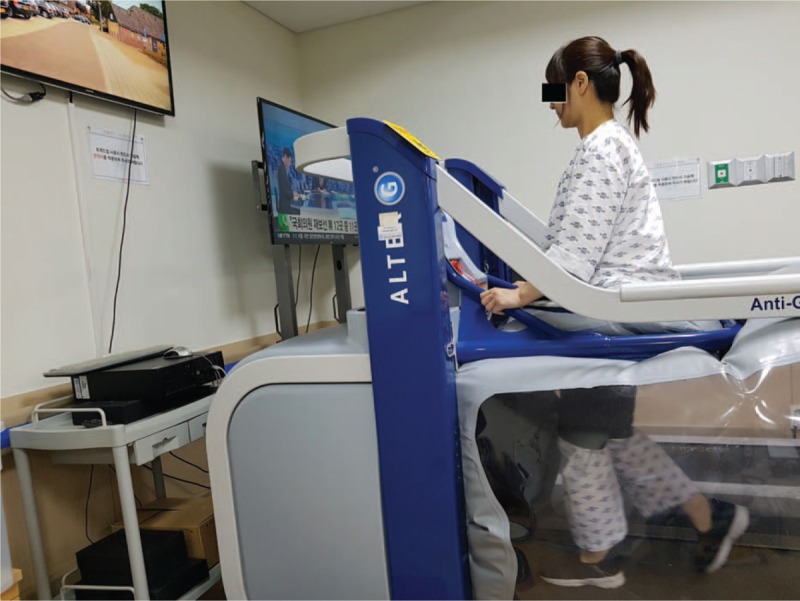
The gait training exercises consisted of Alter G antigravity treadmill (AlterG, Anti-Gravity Treadmill, Fremont, CA).

As an inpatient, the patient received conventional occupational therapy focusing on upper-extremity stretching, strengthening, and performing daily activities for 30 minutes, and physical therapy including lower-extremity muscle strengthening and aerobic exercise for 30 minutes. In addition, the patient received gait training for 30 minutes using a lower-body positive pressure (LBPP) treadmill (AlterG Anti-Gravity Treadmill, Fremont, CA) (Fig. 2)—a medical device that provides air in a pressure-controlled chamber to support body weight. We used a calibration protocol allowing unweighting from 50% to 100% of the patient's body weight in 5% increments, while 100% means that patients do not have to support any of their own weight. Rehabilitation was performed 5 times a week for 1 month.

After 1 month, manual muscle test scores generally improved from grades 1–2 to 3–4, and the ASIA classification improved to C5 incomplete ASIA-D. SCIM improved to 84 due to improvements in independent walking, lower-extremity dressing, and toileting. The Berg balance scale score improved to 50, the 6MWT distance improved to 260 m, and the TUG test time improved to 8.6 seconds. The patient was discharged home and scheduled to attend outpatient rehabilitation twice a week.

## 3 Discussion

Tables 1 and 2 summarized the patients’ improvement in function before and after rehabilitation therapy. The 2 cases presented here indicate the effectiveness of intensive rehabilitation therapy in patients with NMO. The prevalence of NMO is higher in Oriental and Asian populations than in Western populations,^[9]^ but there are few studies on the effects of rehabilitation in patients with NMO in Oriental and Asia. This study is the first to report the rehabilitation effect of NMO patients in the Oriental or Asian region treated with inpatient intensive rehabilitation program.

**Table 1:**
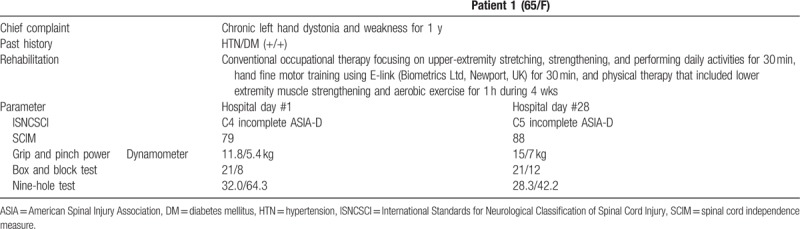
Improvement in function of patient 1 before and after rehabilitation therapy.

**Table 2:**
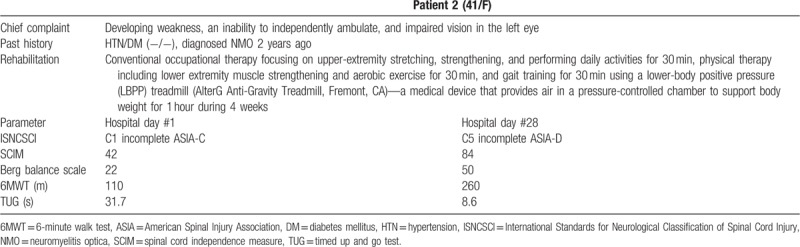
Improvement in function of patient 2 before and after rehabilitation therapy.

Schreiber et al^[14]^ described 3 patients with NMO who were treated in an inpatient rehabilitation unit in United states for 1 to 1.5 months. Although all 3 patients improved, the authors did not state what exercise devices the patients were treated with during rehabilitation. In the current report, both cases were treated using devices (E-link for case 1 and LBPP treadmill for case 2).

The E-link allows standardized pinch and grip measurements, and is a flexible rehabilitation method that can be tailored to specific patient needs in isometric strengthening, motor learning and control, and pinch and grip exercises, and is thus appropriate for a wide spectrum of injuries.^[15]^ Although E-link is a useful tool for assessing hand function,^[16–18]^ there are no studies of its utility for treating dystonia and weakness in patients with musculoskeletal and neurological diseases. This is the only study that has shown an improvement in hand function after using E-link as part of the rehabilitation of a patient with NMO.

The LBPP is a treadmill surrounded by a pressure chamber. The user wears a special pair of shorts that zip at the waist into a pressurized airtight enclosure, which is suspended over the treadmill surface.^[19]^ Although the LBPP is an effective and safe method to improve functional ability in very weak patients with musculoskeletal and some neurological diseases,^[20–23]^ there are no studies showing its effectiveness in patients with NMO. By controlling the pressure in the enclosure, between 20% and 100% of the patient's body weight can be unloaded, in precise 1% increments for low-impact, pain-free movement.^[20]^ The LBPP supports normal walking and running biomechanics, unlike a conventional treadmill, and provides real-time gait data and video monitoring to help patients improve their experience and results.^[23]^ In this study, we showed that rehabilitation that included LBPP training improved lower limb performance and locomotion in a patient with NMO.

Although many studies have reported the effects of rehabilitation on outcomes in individuals with MS,^[10–12]^ few have considered individuals with NMO.^[14,24]^ Nechemia et al's^[24]^ study showed that individuals with NMO had better outcomes after rehabilitation than individuals with MS, regardless of the initial functional status; this was shown to be because they had better cognitive function and communication abilities. Thus, individuals with NMO have better prognosis after rehabilitation than individuals with MS.

The clinical course of NMO is either monophasic or relapsing. The initial presentation of monophasic NMO is more severe than that of relapsing NMO, but recovery is better. Relapsing NMO has a poor prognosis, with more than half of patients developing severe visual loss and an inability to ambulate without aids within 5 years of disease onset.^[5]^

Moreover, it revealed that patients with relapsing NMO are at high risk for cervical myelitis, which can cause respiratory failure and death. In patients with relapsing NMO, such as case 2, we propose that early rehabilitation would minimize complications and improve function.

There are few articles emphasizing the importance of early rehabilitation in patients with NMO; however, many studies in stroke and Parkinson disease have revealed that early intensive rehabilitation is associated with greater and faster improvement in performance of activities of daily living and quality of life.^[25,26]^

This report has several limitations. First, it was a retrospective report of only 2 cases, and the results may not generalize to all patients with NMO. Second, we did not consider the impact of medical treatment of NMO. The impact of medical treatment affected the outcome of the patients presented in this report. Thus, further studies addressing these limitations are warranted.

## 4 Conclusion

An intensive, multidisciplinary rehabilitation program may lead to neurological and functional gains in patients with NMO. Further studies are needed to validate rehabilitation protocols and their effects.

## Author contributions

**Conceptualization:** Won Bin Kim, So Young Lee.

**Data curation:** Won Bin Kim.

**Formal analysis:** Won Bin Kim.

**Investigation:** Won Bin Kim.

**Methodology:** Won Bin Kim, So Young Lee.

**Project administration:** So Young Lee.

**Resources:** So Young Lee.

**Software:** Won Bin Kim.

**Visualization:** Won Bin Kim.

**Writing – original draft:** Won Bin Kim.

**Writing – review & editing:** Won Bin Kim, So Young Lee, Bo Ryun Kim, Youn Ji Kim.
